# Torticollis

**Published:** 1909-05-01

**Authors:** 


### SURGERY.

### TORTICOLLIS.

Cases of torticollis may be divided into two main classes—(*a*) congenital and (*b*) acquired. In the first class the condition exists from birth or from a very early period, while the acquired variety may come on at any age, and the abnormality of the sterno-mastoid muscle may be ascribed to some definite cause. What is the actual cause of the contracture in the congenital cases is not easy to say. It is commonly held that the shortening of the sterno-mastoid is due to an injury at birth which causes a hæmatoma. The blood-clot is invaded by connective tissue cells, which develop into fibrous tissue. This contracts in obedience to its normal function, and the muscle is thereby shortened. This hypothesis has been objected to on two grounds: (1) that in many cases a hæmatoma was formed in the sterno-mastoid at birth, but torticollis did not follow; and (2) that in many cases of torticollis no hæmatoma has been observed. But in order to justify a hypothesis one has not to show that every case obeys the rule, but only a majority; and there are quite enough cases of torticollis in which a hæmatoma was observed to render the suggestion a tenable one. Those who object to the "hæmatoma" theory are inclined to regard malposition in utero and congenital syphilis as causative factors.

The acquired form is due either to rheumatism, which may cause in the first place a myositis of the sterno-mastoid—the so-called stiff neck—and the deformity may become permanently acquired from this small beginning—a kind of habit spasm, as it were. But more commonly cicatricial contraction of the skin resulting from burns etc., or an intra-muscular abscess or gumma, may be the starting-point of the malady.

There is yet a third class of torticollis—the spasmodic. In this the contracture is due to irritation to some part of the nervous arc supplying the muscles of the neck. These cases are more complicated because of the complexity of the responsible cause or causes; and, secondly, because the sterno-mastoid is rarely affected alone, but it is usual to find also contractures of some of the muscles of the posterior triangle of the neck.

Thus the cortical centres of the nerves may be directly irritated, or the nerve trunks themselves may be pressed upon by inflamed glands, or the results of caries of the cervical spine, or the irritation may be reflex, and may be set up by a cause so apparently remote as carious teeth. In addition to the sterno-mastoids, one or other of the following muscles may also be affected: the trapezius, the splenius, the complexus, or the scalenus. In investigating the cause in such a case, caries of the cervical spine should always be eliminated.

In the first two groups of cases the sterno-mastoid is primarily at fault. As the result of its contraction the head is flexed and the chin is tilted to the opposite side and the shoulder is raised on the affected side. But other results follow later: the half of the face on the side of the contracted muscle is generally smaller than the sound side, and the features are less developed. If the condition is allowed to persist secondary contraction of adjacent strands of fasciæ takes place, a point which must be borne in mind in treating the condition, since, if a successful result is to be obtained, these must be divided as well as the muscle which is primarily at fault. It has been argued that the ill-development of the face is due to a central lesion, but that it is more in the nature of a disuse-atrophy is shown by the fact that in cases in which the deformity is remedied by operation at an early age, the maldevelopment tends gradually to correct itself. When the case is examined the sterno-mastoid on the affected side can be felt to stand out as a prominent unyielding structure, more like a fibrous band than a muscle, as, indeed, in most cases it is.

The spasmodic cases differ in the picture presented. The subjects of congenital torticollis rarely complain of anything but the deformity. Sometimes they suffer discomfort from the unnatural position of the head, but most often they become accustomed to this, so that it hardly worries them at all. In the spasmodic cases, however, there is both intermittent spasm and pain, and, in contradistinction to the congenital type the affected muscles are hypertrophied rather than atrophic.

The treatment of the congenital cases is not difficult, and operation will usually improve the condition, although a perfect result is not always obtained. An operation is really the soundest procedure in all congenital cases, although it has been said that a successful result may be obtained by manipulation or mechanical means if the treatment is begun early. But the operation is a simple one, and leaves scarcely any disfigurement, and the treatment after operation is certainly shorter than in a case in which manipulative measures have been tried from the beginning. The operation consists in dividing the sterno-mastoid. This may be done either by subcutaneous tenotomy or by an open operation. The latter is much to be preferred. The only valid argument in favour of tenotomy is that it leaves little or no scar; but against it may be urged the difficulty of being certain that all resisting structures have been divided, and if any single one of them is left the operation will fail in its object. It has also been said that in tenotomy the external jugular vein may be cut, but this is a slight objection. The after-treatment is most important. The whole point of it is to devise some form of retentive apparatus which will keep the head in a rather over-corrected position. The simplest is to apply a plaster of Paris splint, which is made after the manner of a Bavarian splint for the leg, so that it can be taken off daily and massage and passive movement can be applied. In the acquired forms a cure can only be obtained by getting rid of the cause. In contracture due to cicatrisation, a plastic operation, the details of which will depend in each case upon the particular condition found, will probably be necessary; and the after-treatment is the same as that described for the congenital variety.

